# Osteopontin at the Crossroads of Inflammation and Tumor Progression

**DOI:** 10.1155/2017/4049098

**Published:** 2017-07-09

**Authors:** Luigi Mario Castello, Davide Raineri, Livia Salmi, Nausicaa Clemente, Rosanna Vaschetto, Marco Quaglia, Massimiliano Garzaro, Sergio Gentilli, Paolo Navalesi, Vincenzo Cantaluppi, Umberto Dianzani, Anna Aspesi, Annalisa Chiocchetti

**Affiliations:** ^1^Department of Translational Medicine, University of Eastern Piedmont, Novara, Italy; ^2^Department of Health Sciences and Interdisciplinary Research Center of Autoimmune Diseases (IRCAD), University of Eastern Piedmont, Novara, Italy; ^3^SCDU Anestesia e Rianimazione, Azienda Ospedaliero-Universitaria Maggiore della Carità, Novara, Italy; ^4^Department of Translational Medicine, Nephrology and Kidney Transplant Unit, University of Eastern Piedmont, Novara, Italy; ^5^Department of Health Sciences, University of Eastern Piedmont, Novara, Italy; ^6^Department of Surgery, University of Eastern Piedmont, Novara, Italy; ^7^Anestesia e Rianimazione, Università “Magna Graecia” di Catanzaro, Catanzaro, Italy

## Abstract

Complex interactions between tumor and host cells regulate systemic tumor dissemination, a process that begins early at the primary tumor site and goes on until tumor cells detach themselves from the tumor mass and start migrating into the blood or lymphatic vessels. Metastatic cells colonize the target organs and are capable of surviving and growing at distant sites. In this context, osteopontin (OPN) appears to be a key determinant of the crosstalk between cancer cells and the host microenvironment, which in turn modulates immune evasion. OPN is overexpressed in several human carcinomas and has been implicated in inflammation, tumor progression, and metastasis. Thus, it represents one of the most attracting targets for cancer therapy. Within the tumor mass, OPN is secreted in various forms either by the tumor itself or by stroma cells, and it can exert either pro- or antitumorigenic effects according to the cell type and tumor microenvironment. Thus, targeting OPN for therapeutic purposes needs to take into account the heterogeneous functions of the multiple OPN forms with regard to cancer formation and progression. In this review, we will describe the role of systemic, tumor-derived, and stroma-derived OPN, highlighting its pivotal role at the crossroads of inflammation and tumor progression.

## 1. Inflammation and Cancer

Inflammation is a physiological response of the body aimed to remove harmful stimuli, including damaged cells, irritants, pathogens, and sterile injuries, such as cancer, and then to begin the healing process. Several proinflammatory cytokines released in the first steps of inflammation can induce the activation of regeneration-promoting pathways. Among the downstream signals, IL-6 can activate proregenerative transcription factors, such as YAP, STAT3, and Notch [1]. During inflammation, fibroblast recruitment and fibrosis are frequently observed. Fibroblasts produce collagen and other extracellular matrix components in the tumor microenvironment thus stimulating cancer cell proliferation and angiogenesis.

Myeloid cells, including macrophages and neutrophils, are the first immune cells involved in inflammation and are abundant in the tumor microenvironment [2, 3]. Tumor-associated macrophages (TAMs) play a key role in cancer development and progression through stimulation of cell survival and proliferation, angiogenesis, invasiveness and motility, and suppression of CTL responses [4]. During the early stages of tumor development, TAMs seem to acquire a classical activated M1 phenotype secreting proinflammatory mediators, such as IL-1, IL-6, TNF*α*, IL-23, and iNOS, which are involved in tumor initiation [5]. During tumor progression, molecules produced by immune and cancer cells, such as IL-4, CSF1, and TGF-*β*, contribute to the switch of TAMs to the alternative M2 phenotype-producing anti-inflammatory and proangiogenic molecules, such as IL-10, arginase 1, TGF-*β*, and vascular growth factor (VEGF) supporting the tumor growth [4]. Moreover, in this phase, fibroblasts can secrete several cytokines, chemokines, and other molecules, including osteopontin (OPN) [6, 7].

## 2. Osteopontin (OPN) in Inflammation

Osteopontin (OPN) is an extracellular matrix protein also referred to as bone sialoprotein 1 (BSP-1), secreted phosphoprotein 1 (SPP1), and early T lymphocyte activation 1 (ETA-1). This plurality of names reflects the involvement of OPN in multiple physiological and pathological processes [8, 9].

**Figure 1:**
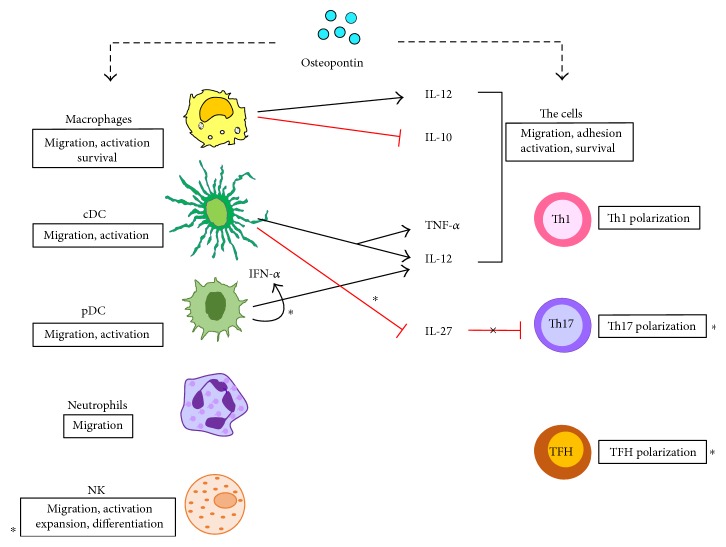
Effect of OPN on several leukocytes. sOPN triggers myeloid and lymphoid cells eliciting a functional response (boxes) that in turn induces cytokine secretion which drives the inflammatory/immune response. Asterisks mark the effects mediated by iOPN.

The main role of OPN during inflammation is to trigger different leucocytes eliciting a functional response and inducing cytokine secretion, in order to shape the entire immune response (Figure 1).

### 2.1. Macrophages

As an integrin-binding protein, OPN primarily not only stimulates migration, accumulation, and retention of macrophages at sites of injury but can also modulate their cytokine production by promoting Th1 cell-mediated immunity and stimulating their differentiation from monocytes. OPN controls several immune cell functions including monocyte adhesion, migration, differentiation, and phagocytosis [10].

Migration of macrophages is influenced by interaction of OPN with *α*_4_ and *α*_9_ integrins, but a role is played also by its interaction with CD44. Moreover, OPN inhibits macrophage apoptosis by interacting with *α*_4_ integrin and CD44 [11, 12].

OPN stimulates IL-12 and inhibits IL-10 production at sites of inflammation in macrophages, with a strong proinflammatory effect [13]. Wound healing studies in mice showed that OPN is expressed at high levels in infiltrating leukocytes during the acute phase of inflammation and regulates leukocyte infiltration and activation as well as tissue remodeling. Remarkably, OPN downregulation at the site of wound reduced macrophage infiltration and enhanced wound healing [14].

Furthermore, OPN induces the expression of matrix metalloproteinases (MMP), in particular MMP-2 and MMP-9, which are involved in matrix degradation, cell migration, and tissue remodeling [15]. OPN activates the transcription factors AP-1 and NF-*κ*B thus regulating the production of inflammatory mediators during cell-mediated immunity. For example, OPN induces PI3-kinase-dependent Akt phosphorylation and enhances the interaction between phosphorylated Akt and IKK*α*/*β* through the engagement of CD44 and *α*v*β*3 integrin. Moreover, OPN increases NF-*κ*B activation through phosphorylation and degradation of I*κ*B*α* by inducing the IKK*α*/*β* activity [16].

### 2.2. Dendritic Cells (DCs)

OPN is involved in conventional DC migration by interacting with CD44 and *α*_v_ integrin [17]. OPN is expressed at a higher level in immature DCs than in mature DCs; thus, it was suggested that OPN acts as an autocrine and/or paracrine signal for DC maturation [18]. Moreover, OPN acts as a prosurvival signal for DCs since OPN blockade results in their reduced expression of costimulatory and MHC class II molecules and increased apoptosis [18]. Following activation by OPN, DCs produce IL-12, in a CD40 ligand- and IFN-*γ*-independent manner, and increase expression of MHC class II molecules, CD80/CD86, and ICAM-1, which enhances the their Th1-polarizing ability [19]. Intriguingly, an intracellular form of OPN (iOPN, see below) inhibits IL-27 expression in conventional DCs and enhances their ability to promote proinflammatory T helper type 17 (Th17). In plasmacytoid DC (pDC), iOPN supports IL-12 secretion and promotes IFN*α* expression through interferon regulatory factor (IRF) 7 activation.

### 2.3. T Cells

OPN is involved in Th cell polarization by enhancing Th1 and Th17 differentiation and inhibiting Th2 cytokine expression. By interacting with CD44 in Th cells, OPN induces hypomethylation of *IFN-γ* and *IL-17α* genes enhancing production of IFN-*γ* and IL-17A. Moreover, CD44 deficiency promotes hypermethylation of *IFN-γ* and *IL-17α* and hypomethylation of *IL-4* gene, leading to Th2 cell differentiation [20]. Recent data detected a key role of iOPN in T follicular helper (TFH) differentiation.

### 2.4. Neutrophils

It has been shown that OPN acts on neutrophil recruitment but has no influence on their phagocytic activity and superoxide, cytokine, and MMP-9 production [21]. In vitro assays showed that the RGD sequence in OPN is required for neutrophil migration [22] and OPN-induced neutrophil migration is dependent on ERK and P38 MAP kinases activation [23]. By contrast, the OPN interaction with CD44 seems to play a minor role in neutrophils [21].

### 2.5. Natural Killer

OPN plays a key role in increasing NK cell migration and activation. In a mouse model of ischemia- and reperfusion-induced kidney injury, OPN has been shown to be involved in NK cell-mediated tubular epithelial cell apoptosis [24]. IL-15 induces iOPN expression in NK cells [25], which results in increased mTOR activity leading to NK cell expansion and differentiation. Moreover, iOPN seems to play a role in differentiation of long-lived NK cells with a memory-like phenotype following homeostatic expansion [26].

## 3. OPN in Cancer

OPN is involved in multiple physiological and pathological processes, starting from inflammation. In particular, OPN plays a key role in cancer progression by enhancing proliferation, survival, motility, and invasion of tumor cells in breast cancer, hepatic carcinoma, prostate cancer, colorectal cancer, lung cancer, and melanoma [27–33]. Overexpression of OPN has been detected at the tumor sites and in the blood of patients, and its levels correlate with tumor stage and aggressiveness, suggesting that OPN can be a diagnostic and prognostic biomarker for several cancers [29].

OPN is expressed by many cell types such as bone cells (e.g., osteoblasts, osteoclasts, and osteocytes), immune cells (e.g., T cells, B cells, natural killer cells, and macrophages), neural cells, epithelial cells, fibroblasts, smooth muscle cells, and endothelial cells [34–36] and in several tumor-derived cell lines. It is distributed in a variety of tissues and is secreted in body fluids including blood, urine, bile, and milk.

## 4. OPN General Features

This plurality of OPN isoforms, due to polymorphisms in its gene, posttranslational modifications (PTMs), and binding partners, reflects its broad functions.

### 4.1. OPN Gene

OPN is a highly acidic protein encoded by the *SPP1* gene, located in chromosome 4 (4q22.1). The gene comprises 7 exons with the translation start codon in exon 2. A large number of polymorphisms can be found scattered throughout the gene and a few of them have been associated with a higher risk of developing autoimmune diseases and cancer [37, 38]. In autoimmune diseases, risk alleles of OPN may support production of high levels of OPN and cooperate with other alterations such as apoptosis defects [39–48]. The expression of OPN is influenced by genetic polymorphisms in its promoter [49] and by the presence of several types of regulatory and transcription factor-binding sequences. One of the most studied OPN single-nucleotide polymorphism is rs11730582, whose −443 CC genotype has been associated with higher expression of OPN and increased cancer risk in acute myeloid leukemia, glioma, and papillary thyroid cancer. Interestingly, in other tumors such as hepatocellular carcinoma (HCC), breast cancer, nasopharyngeal carcinoma, and melanoma, the −443 TT genotype, rather than the C allele, correlates with increased expression of OPN [50–56]. In this regard, it has been proposed that the proto-oncogene c-Myb mediates induction of OPN expression levels from the C allele in some tumors, whereas in other malignancies, a yet unidentified transcription factor could activate transcription of OPN from the T allele [56]. Other individual polymorphisms and haplotypes in the promoter region were reported to affect gene expression [57], while variants in the 3′ UTR may affect RNA stability and lead to altered protein levels.

OPN gene expression is modulated by several cytokines (e.g., IL-1*β*, IL-6, TNF-*α*, and IFN-*γ*), hormones (e.g., vitamin D, estrogen, angiotensin II, and glucocorticoids), platelet-derived growth factor, and oxidized low-density lipoprotein [58].

### 4.2. sOPN

The protein exists in a myriad of different isoforms due to alternative splicing and a number of posttranslational modifications (PTMs), such as serine/threonine phosphorylation, glycosylation, tyrosine sulfation, and proteolytic cleavage. OPN consists of 314 amino acid residues, which confer a predicted molecular weight of 35 kDa; however, because of splicing and PTMs, the actual molecular weight ranges from 41 to 75 kDa [59]. OPN-a represents the full-length isoform, but two other splice variants can be found: OPN-b, which lacks exon 5 and OPN-c, which lacks exon 4. Exon 5 contains a cluster of phosphorylated serine/threonine residues [60] (Table 1). Since exon 4 contains the target sequence for transglutaminase, OPN-c, unlike the other isoforms, cannot form polymeric complexes [61].

OPN isoforms have often distinct expression profiles and different biological effects. For example, an anti-OPN-exon4 antibody, able to recognize both OPN-a and OPN-b isoforms, stained exclusively the cytoplasm of breast cancer cells, while OPN-c was predominantly detected in their nucleus [62]. Given that nuclear internalization signals are common to all OPN splice variants, the reason why OPN-c was selectively expressed in the nucleus still remains to be determined. Furthermore, OPN-c was detected in breast carcinoma but not in normal surrounding tissues [63], whereas OPN-a and OPN-b expression levels were found in both tissues. Granted that enhanced expression of OPN-c levels in breast cancer cells correlates with tumor grade and poor prognosis [64] and OPN-c is highly expressed in pancreatic and colon cancers [65], it has been proposed that this isoform may constitute a potential prognostic factor for these carcinomas. OPN-c is also an efficient biomarker able to distinguish prostate cancer from benign prostate hyperplasia, with a sensitivity of 90% and a specificity of 100% [66]. In addition, OPN-c can also promote proliferation and migration of ovarian cancer cells [67]. Other studies have shown that OPN-a is the predominant form in a number of lung cancer, liver cancer, papillary thyroid carcinoma, and mesothelioma specimens [68–71]. Furthermore, HCC predominantly expressed OPN-a and OPN-b, and these isoforms, unlike OPN-c, could induce cell migration [70]. Lastly, in glioma cells, only OPN-a and OPN-c but not OPN-b were able to promote invasiveness [72]. Thus, the expression patterns and functions of OPN-splicing isoforms appear to be tumor specific and in some cases clinically relevant.

### 4.3. iOPN

Besides its secreted form, OPN can be found in its intracellular form (iOPN), which is a truncated version of the full-length protein lacking the signal sequence due to initiation of translation from a downstream noncanonical start codon [73]. The biological functions of iOPN are mainly related to the regulation of cytoskeletal rearrangement and signal transduction pathways [50]. iOPN was found in dendritic cells [73–75], macrophages [76], and nerve cells [77]. Indeed, iOPN localizes to the nucleus of 293 cells where it mediates cell duplication through association with polo-like kinase 1 [54], whereas in fibroblasts, iOPN plays a role in cell migration [78]. Moreover, deficient expression of iOPN in natural killer (NK) cells causes impaired expansion and increased apoptosis of these cells following stimulation with IL-15, resulting in defective immune response to viral infection and tumor cells [79]. In pDC, iOPN mediates Toll-like receptor 9 (TLR-9) signaling and enhances IFN-*α* production through the interaction with myeloid differentiation primary response gene 88 (MyD88) [75]. Under stimulation of cellular debris released by necrotic hepatocytes, iOPN inhibits the activation of TLR/MyD88 signaling in macrophages through interaction with MyD88, acting as a negative regulator of TLR-mediated immune responses [79]. In follicular T helper (TFH) cells, iOPN is involved in signaling through ICOS, a costimulatory receptor involved in T cell function [80–83]. Upon ICOS triggering, iOPN interacts with the PI3K p85*α* regulatory subunit, translocates into the nucleus, and binds Bcl-6 (involved in TFH differentiation) protecting it from proteasome-mediated degradation.

### 4.4. PTMs

**Figure 2:**
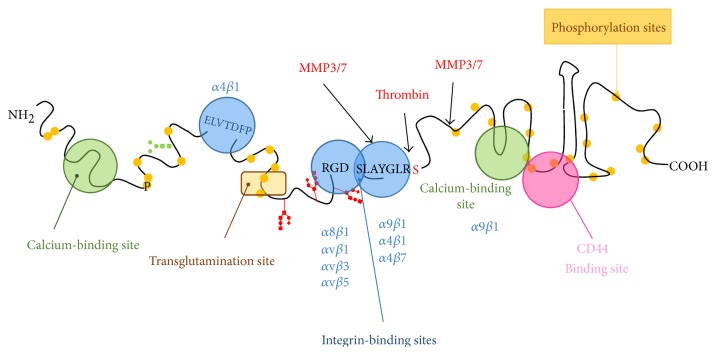
Main domains of OPN. The cartoon depicts the functional parts of OPN. OPN binds two different classes of receptors, integrins (in blue) and CD44 (in pink). It can also interact with calcium (green). The SVVYGLR sequence is usually masked in the full-length molecule, but it becomes available upon thrombin cleavage of OPN. OPN undergoes several posttranslational modifications including glycosylation (red and green sugars), phosphorylation (yellow dots), crosslinking mediated by transglutaminase and protease cleavage (thrombin and MMPs). Each of these modifications can alter OPN functions.

The presence of different isoforms and the heterogeneity due to the large number of PTMs can partly account for the multiplicity of functions ascribed to OPN (Figure 2). Another important aspect is the ability of OPN to interact with different receptors. Indeed, OPN contains an Arg-Gly-Asp (RGD) sequence, which binds to integrins such as *α*v*β*1, *α*v*β*3, *α*v*β*5, *α*8*β*1, and *α*5*β*1 [84]. The adjacent Ser-Val-Val-Tyr-Gly-Leu-Arg (SVVYGLR) sequence is a cryptic motif that is exposed upon cleavage by thrombin and interacts with *α*4*β*1, *α*9*β*1, and *α*4*β*7 integrins [85], which are present on the surface of immune cells such as T cells, neutrophils, macrophages, and mast cells. Thrombin cleavage produces an N-terminal fragment (OPN-N) that contains the aforementioned integrin-binding domains and a C-terminal fragment (OPN-C) that presents a binding site for specific splice variants of CD44. Moreover, the C-terminal fragment produced in mouse after cleavage by MMP-3 and -7 can bind to *α*9*β*1 integrin through the LRSKSRSFQVSDEQY cryptic motif [86]; however, this interaction does not occur with the uncleaved protein. Apart from thrombin and MMPs, OPN can also be a substrate for plasmin and cathepsin D and contains binding sites for calcium and heparin [87]. Interestingly, OPN variants produced by enzymatic cleavage can retain their activity or acquire additional functions. In this regard, N-terminal fragments have shown a greater capability to mediate RGD-dependent cell attachment than the full-length protein, presumably because of a more exposed integrin-binding sequence [88]. In the bone marrow, the predominant OPN form is the N-terminal fragment produced by thrombin cleavage. This fragment acts as a chemotactic factor for hematopoietic stem cells through interaction with *α*9*β*1 and *α*4*β*1 integrins after transplantation [89]. In T cells, OPN-N induces IL-17 upregulation, while OPN-C induces IL-10 downregulation, and each fragment has specific effects on cell adhesion and migration [90]. Cleavage of OPN-c by MMP-9 in HCC produces fragments that enhance cellular invasion and appear to correlate with the HCC metastatic potential [91]. Inhibition of thrombin in breast cancer cells that express OPN decreases tumor growth and metastasis [92]. Lastly, bioactive OPN fragments can also be generated after processing of sOPN by extracellular proteasomes, resulting in the production of fragments with new chemotactic activity, which may be relevant for cancer progression since high levels of both OPN and extracellular proteasome can be detected in the tumor mass [93].

## 5. Systemic OPN Levels in Cancer

OPN is a ubiquitously expressed protein whose secreted form exerts pleiotropic effects on a number of fundamental biological processes, such as proliferation, apoptosis, bone formation, and angiogenesis [94–96]. Despite its importance in such processes, several different studies addressing the role of OPN in tumors have led to conflicting results. In recent years, in an effort to establish common denominators, several meta-analyses of multiple studies on high-incidence cancers worldwide have been carried out. These tumors included colorectal cancer (CRC), malignant pleural mesothelioma (MPM), ovarian cancer, non-small-cell lung carcinoma (NSCLC), breast cancer, gastric cancer, and HCC [97–101]. The following is a comprehensive survey of those studies.

In a selection of 10 clinical cohort studies represented by a total of 1133 NSCLC patients, Shi et al. showed a shorter overall survival in OPN-positive patients compared to that in OPN-negative patients with no significant differences between the Asian and the Caucasian groups, thus suggesting a role for OPN as a prognostic factor for NSCLC [102]. Moreover, the analysis by Hu et al. of a cohort of six studies, with a total of 906 participants, of which 360 were MPM patients and 546 healthy individuals, showed that the diagnostic accuracy of OPN for MPM was comparable to that of the soluble mesothelin-related peptides (SMRP), the most promising serum biomarker for MPM [103]. On the other hand, a meta-analysis of 8 studies published up to February 2014 with clinical cohorts ranging from 100 to 333 breast cancer patients [104] showed no correlation between OPN expression and several other diagnostic/prognostic biomarkers commonly used for breast cancer, such as HER2, PR, and ER, although a significant association between OPN overexpression and both lymph node metastasis and overall survival was reported. A more recent meta-analysis of 10 studies with a total of 1567 patients, extrapolated from papers published up to December 2015, evaluated the role of OPN-splicing variants [105]. In good agreement with Xu et al. [104], OPN overexpression correlated with poor overall survival. In particular, the authors found that OPN-c but not OPN-a was specifically expressed by breast cancer cells and that the correlation between poor overall survival in breast cancer and OPN-c overexpression was more statistically significant than that obtained with full-length OPN [105]. In a meta-analysis of 15 studies with a cohort of 1698 CRC patients, Zhao and coworkers reported a significant correlation between OPN expression and lymph node metastasis or tumor-distant metastasis. However, the authors did not find any statistical correlation between OPN expression and tumor invasiveness. Moreover, at 2, 3, and 5 years after diagnosis, the overall survival of patients with high expression of OPN was significantly reduced, indicating that OPN is a valid prognostic marker for CRC [101]. Another study by Wang and colleagues, who analyzed 15 studies involving a total of 1653 subjects, of which 822 were ovarian cancer patients and 831 healthy individuals, found positive association between OPN levels in the serum and ovarian neoplasm, with differences in ethnicity and higher association in the Asian versus the Caucasian group. Intriguingly, the authors speculated that high serum levels of OPN could induce tumor survival and proliferation through inhibition of the proapoptotic PI3-K/Akt signaling pathway, concluding that higher serum levels of OPN may correlate with the aggressive progression of ovarian neoplasm. Thus, according to this study, OPN levels in the bloodstream can be used both as diagnostic and prognostic markers for ovarian neoplasms [106].

Even though there are no meta-analysis results published on melanoma, an aggressive skin cancer whose incidence has been stably increasing [107–109], several studies have been published to elucidate the role of OPN in melanoma and uveal melanoma progression [110–112]. All these studies have shown that OPN overexpression is strongly associated with reduced overall survival of melanoma patients. In addition, Filia and colleagues reported enhanced plasma OPN levels in stage IV melanoma [112]. Finally, Barak et al. showed a remarkable increase in OPN expression levels in uveal melanomas several months before the diagnosis of a metastatic phenotype [113].

**Figure 3:**
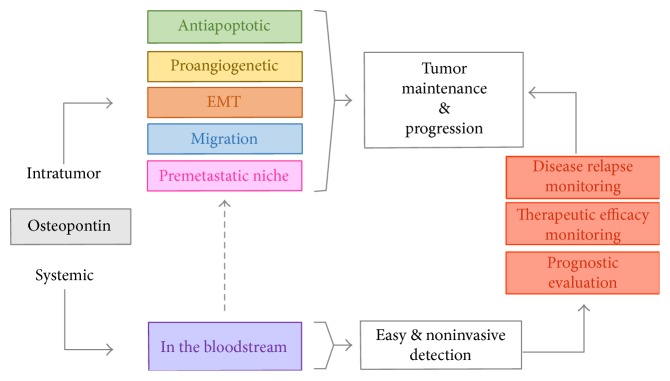
Schematic representation of the role of OPN in tumor patients. OPN is involved in many different biological functions as well as in tumor maintenance and progression. Besides its local effect, OPN is also secreted in the blood stream, and its levels are increased in patients with different tumor types. Since measurement of OPN from plasma or serum is readily accessible and noninvasive, it is likely that OPN might become a useful marker for the diagnosis, treatment, and tumor relapse monitoring of a number of carcinomas.

Taken all together, the aforementioned studies clearly point to a key role of OPN in tumor progression, thereby paving the way for future clinical applications (Figure 3). These include (i) the use of OPN as a prognostic biomarker for different tumors and its use as a predictor of therapeutic efficacy, (ii) the design of OPN-based targeted therapy specific for different tumor stages to improve treatment efficacy, and (iii) the use of OPN to monitor the occurrence of disease relapse during follow-up, since blood sampling is a fast, minimally invasive, and readily repeatable procedure.

Overall, given the potential of OPN use in clinics, the development of common procedures for sample collection and OPN measurement could become the standard of care and prevention of malignant primary tumors.

## 6. OPN^−/−^ Mice

The pathophysiological role of OPN on tumor enhancement/progression as well as on immune system has been investigated in different studies using in vivo models. Solid tumors are composed by genetically mutated cancer cells dispersed into a stroma formed by a variety of normal cells and extracellular matrix (ECM). Since the stroma actively participates in tumor progression, including the metastatic process, bidirectional communication between tumor cells and the associated stroma strongly affects disease initiation, progression, and patient prognosis [114].

For this reason, in tumor-induced models of cancer (i.e., breast, melanoma), in order to dissect the individual role played by stroma-secreted versus tumor-secreted OPN, the use of OPN^−/−^ mice is preferred. In this setting, comparison among OPN^+/+^ and OPN^−/−^ mice will give insights into the contribution of stromal OPN (i.e., missing in OPN^−/−^). Knocking down OPN into the tumor cells, will highlight the contribution of tumor OPN.

In general, it has been described that leucocyte recruitment at the site of inflammation, mainly neutrophil infiltration and macrophage accumulation, is impaired in OPN^−/−^ mice with concomitant inhibition of proinflammatory cytokine release [10, 115, 116]. OPN regulates immunosuppression at tumor sites by favoring the presence of immunosuppressive leukocytes at metastatic sites. Sangaletti et al. showed that myeloid-derived suppressor cells (MDSCs) in OPN^−/−^ mice showed lower expression in arginase-1, anti-phospho-STAT3, and IL-6 and were less immunosuppressive compared to those in wild-type controls after injection of 4T1 cell line. In addition, less regulatory T cells accumulated at metastatic site (lung metastasis) in OPN^−/−^ mice and only MDSCs from wild-type mice were able to promote metastasis [117]. Similarly, Kale et al. showed the involvement of OPN in macrophage recruitment into tumor in a mouse model of melanoma. They observed an accumulation of OPN- and cyclooxygenase-2- (COX-2-) positive macrophages at the site of tumor; with increased angiogenesis and melanoma growth and in OPN^−/−^, they reported a strong suppression in tumor growth compared to that of their wild-type counterpart [118]. In another study, Lee and colleagues investigated the role of OPN in promoting liver tumor in a mouse model of diethylnitrosamine- (DEN-) induced hepatic carcinogenesis. In this work, they described a significant reduction in the overall incidence of hepatic tumors in OPN^−/−^ mice compared to that in wild-type mice and they observed that in vitro OPN suppression in human hepatocellular carcinoma cells promoted cell death by apoptosis [119]. A similar observation was reported by Hsieh et al. in a mouse model of squamous papilloma describing that deficiency of OPN resulted in apoptosis and delayed the development of tumor [120].

Altogether, these studies suggest that OPN plays a central role in immune modulation and tumor progression by inducing the expression of mediators and by finely orchestrating the recruitment of cells in order to create an immunosuppressive and protumorigenic microenvironment.

## 7. OPN within Solid Tumors

Tumor development and progression require a suitable microenvironment where tumor cells influence normal resident cells, such as fibroblasts and endothelial cells, while recruiting accessory cells from the bone marrow to initiate angiogenesis [121].

The ECM is produced by both tumor and stromal cells and is composed of structural and functional proteins. The latter ones are collectively referred to as matricellular proteins, which, under physiologic conditions, modulate several cellular processes, including cell adhesion and migration, ECM deposition, cell survival, and proliferation [122]. All these processes are also required for primary tumor growth and metastasis, where matricellular proteins are often aberrantly expressed.

**Figure 4:**
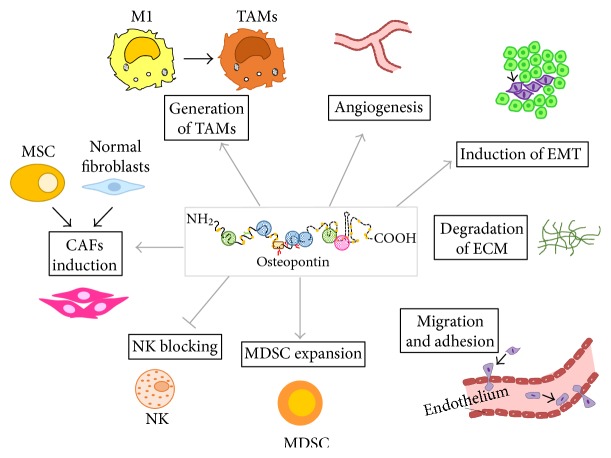
OPN functions in the tumor microenvironment. In the tumor microenvironment, OPN has different functions ranging from the recruitment of leucocytes, endothelial cells, and mesenchymal stem cells (MSCs) from the periphery or bone marrow, to the reprogramming of local fibroblast to cancer-associated fibroblasts and transformation of M1 antitumorigenic macrophages to tumor-associated macrophages. These changes in the stroma favor tumor progression through angiogenesis, degradation of the extracellular matrix, epithelial to mesenchymal transition (EMT), and migration of metastatic cells.

The presence of a tumor leads to stromal changes including the recruitment of leucocytes, endothelial cells, and mesenchymal stem cells (MSCs). It also causes reprogramming of local fibroblasts to cancer-associated fibroblasts (CAFs). These cells secrete growth and matrix remodeling factors and support angiogenesis and lymphangiogenesis to promote further tumor growth and metastasis. Stroma formation in neoplastic processes represents both the host's reaction to neoplastic cells and the ability of tumor cells themselves to modify their environment to influence growth and progression [123, 124]. OPN can be produced by many cell types present in the tumor microenvironment, including the tumor itself. Several functions of OPN have yet been elucidated; however, the role of OPN in tumor growth and progression as well as its contribution to the tumor microenvironment is still partially understood (Figure 4).

Systemic tumor dissemination is regulated by complex interactions between tumor and host cells during the migration of tumor cells into the blood and/or lymphatic vessels at the primary tumor site. These cells subsequently spread to target organs and are capable of surviving and growing at distant sites. In this context, genetic and epigenetic alterations of tumor cells appear to be the key determinants of the crosstalk between cancer cells and the host microenvironment, which in turn modulates intercellular communication, immune evasion, and sustained proliferation.

As described above, OPN is a matricellular protein implicated in inflammation, tumor progression, and metastasis [125] and is found overexpressed in a variety of human carcinomas, including breast, lung, colorectal, stomach, and ovarian, as well as melanoma [125, 126]. Elevated tumor and plasma levels of OPN have been associated with poor prognosis and with reduced survival in patients with breast cancer [64]. OPN is one of the highest expressed genes in a large percentage of patients with glioblastoma [22], and the depletion of OPN in glioblastoma-initiating cells leads to the loss of their tumorigenic potential [127]. Within a tumor mass, the functional activities of OPN are complex, since OPN is generally expressed by both tumor and stroma cells in its secreted form. Moreover, both tumor and normal cells have receptors able to bind with sOPN. This scenario becomes quite complex when we consider that, in some tumor types, OPN is also part of the extracellular matrix. Thus, sOPN produced by tumor cells can influence cells in the tumor microenvironment and vice versa. Whether tumor-derived OPN differs, structurally or functionally, in its effects from stromal-derived OPN still remains to be clarified. Although there is evidence that different OPN isoforms and posttranslational modifications (i.e., phosphorylation, sialation, proteolytic cleavage, transglutaminase crosslinking, and proteolytic processing) may affect different OPN functions [59, 128–130], there is still very scant literature on how tumor-derived OPN may differ from stroma-derived OPN, either structurally or functionally. Interestingly, the distribution of OPN staining may change according to the cancer type and tumor stage. Indeed, in many tumors, OPN^+^ cancer cells are often found at the periphery of invasive tumors adjacent to stromal cells, suggesting its involvement in paracrine tumor and host cellular interactions [131, 132]. Nevertheless, it has been shown that, at least in some instances, tumor-derived OPN is more soluble and not incorporated into the extracellular matrix [133]. Thus, it is unclear whether it is tumor- or stromal-derived OPN (or both) that can be incorporated into the extracellular matrix and affect tumor growth and progression.

In the following two sections, we will explore the documented functions of tumor-derived OPN as well as stromal-derived OPN and OPN function in ECM.

## 8. Effect of Tumor-Derived OPN on the Tumor Microenvironment

Several reports have demonstrated that tumor cells can synthesize and secrete OPN, in vivo. OPN expression in tumor cells has been detected in several cancers [134, 135], including breast, colon, liver, and lung as well as squamous cell carcinomas, as they become aggressive [31, 97, 136–139]. For example, in breast cancer, OPN expression starts increasing as the cancer cells become more aggressive, and knocking down expression of endogenous OPN reduced invasive behavior and suppressed tumor growth in immunocompromised mice [140]. OPN-mediated increase in migration, motility, and invasion have been related to enhanced expression of integrins and CD44 cell surface receptors and to the increase in Met activity [141–143]. Moreover, the first evidence that OPN can cause degradation of a tumor suppressor protein comes from the observation of an inverse correlation between OPN and the tumor suppressor Merlin, in breast cancer [144, 145]. This is the consequence of OPN signaling on the AKT pathway that targets Merlin for ubiquitin degradation.

**Figure 5:**
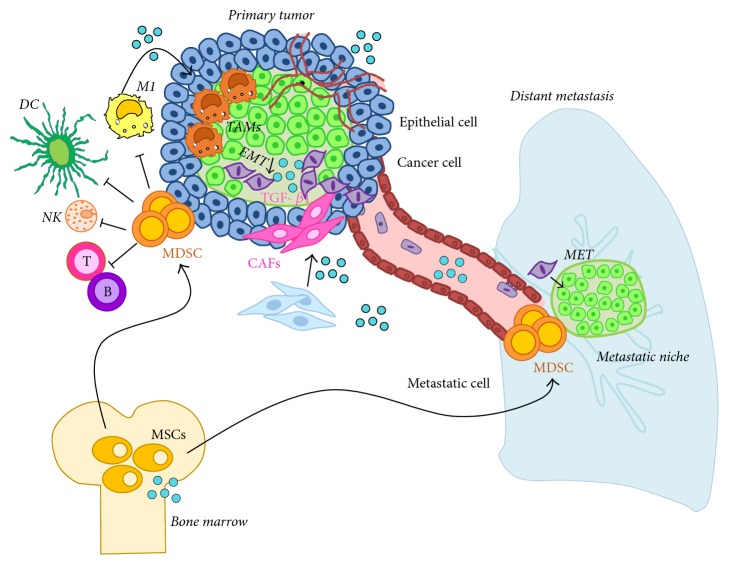
Tumor-promoting functions of OPN. OPN (blue sphere) induces increased proliferation and survival of tumor cells; it is also associated with the recruitment of myeloid-derived suppressor cells (MDSC), from bone marrow stem cells. MDSC create an immunosuppressive niche both in the primary tumor and in the metastatic tissue, thereby creating a favorable place for tumor growth. OPN favors dissemination and angiogenesis to counteract hypoxia, and concomitantly reprograms tissue fibroblasts to cancer-associated fibroblasts (CAFs), which by secreting TGF-*β*1, promote epithelial to mesenchymal transition (EMT) allowing tumor cells to detach from the primary mass and disseminate to the premetastatic niche. Here, OPN promotes mesenchymal to epithelial transition (MET) favoring the metastatic processes.

The tumor-promoting functions of tumor-derived OPN will be reviewed in the following chapters and include (1) increased survival of tumor cells often associated with recruitment of leucocytes at the tumor site; (2) angiogenesis to contrast hypoxia and favor dissemination [139]; (3) reprogramming of tissue fibroblast to CAFs to induce epithelial to mesenchymal transition **(**EMT), thereby allowing tumor cells to detach from the primary mass and disseminate to generate the premetastatic niche [146]; (4) mesenchymal to epithelial transition (MET), that is considered to be the opposite of EMT at the distant site [147] (Figure 5).

### 8.1. Recruitment of Leucocytes at the Tumor Site

#### 8.1.1. Tumor-Associated Macrophages (TAMs)

Macrophages are versatile cells which can be either immunostimulatory or immunosuppressive, thereby promoting or counteracting inflammation, respectively [148, 149]. TAMs are the predominant stromal cell type within the tumor mass. High levels of TAMs often correlate with the advanced tumor stage and poor disease outcome [150]. Several studies have highlighted a causal link between TAMs and neoplastic progression, including tumor initiation, proliferation, immunosuppression, angiogenesis, and metastasis. In the tumor mass, TAMs release cytokines and growth factors that target both tumor and endothelial cells and concomitantly secrete proteases that promote ECM degradation. This never ending process of stroma remodeling favors the release of matrix-bound growth factors and promotes tumor cell motility and invasion [151]. Consistently, TAMs secrete many growth factors essential for neoangiogenesis and tumor proliferation [152, 153].

In a milestone study by Ashkar et al., OPN was shown to act against viral and bacterial infection by inducing a M1 response through upregulation of IL-12 and downregulation of IL-10 [154]. Since OPN exists at least in two forms, depending on its phosphorylation state [130], phosphorylated OPN binds to cell surface receptors (i.e., integrins and CD44), while nonphosphorylated OPN binds to the ECM—the authors went on by showing that while OPN phosphorylation is required to induce integrin-mediated IL-12 production; it is dispensable for CD44-mediated inhibition of IL-10 in macrophages. Interestingly, this mechanism seems to be altered in OPN-mediated immune response in tumors.

In addition to regulating macrophage activation, tumor-derived OPN is also able to attract macrophages to a tumor site by promoting chemotaxis. Using a mouse model of melanoma, Kale et al. demonstrated that TAMs infiltration was significantly reduced in OPN^−/−^ mice compared to that in OPN^+/+^ mice [118], and tumor growth and angiogenesis were significantly reduced in OPN^−/−^ mice compared to that in OPN^+/+^ mice [118]. A more detailed analysis showed that OPN could induce cyclooxygenase- (COX-) 2 expression in macrophages, promoting prostaglandin (PG) E2 production and melanoma cell migration. Furthermore, OPN-mediated *α*9*β*1 integrin activation was required for COX-2 expression and subsequent p38 and ERK activation, which in turn led to increased expression of PGE2 and MMP-9. It seems that OPN inhibits macrophage functions, at least in part, through downregulation of inducible nitric oxide synthase and therefore nitric oxide production, resulting in inhibition of tumor cell death [155–159]. OPN can also have an antitumor activity thanks to its ability to activate type I NK T cells [160] and to inhibit their apoptosis via binding to and activation of the CD44 receptor [161]. This observation strongly suggests that OPN can either inhibit or promote leukocyte functions and that it is the balance between the OPN-elicited macrophage and type I NK T cell response that determines whether the overall effect of the inflammatory response will be tumor promoting or tumor inhibiting [162]. This dual role of OPN will be further discussed later in this review.

#### 8.1.2. Myeloid-Derived Suppressor Cells (MDSCs)

Extramedullary myelopoiesis, that is myelopoiesis occurring outside the bone marrow, including the spleen, is a novel OPN-driven process recently described [163–165]. Extramedullary myelopoiesis may induce the accumulation of peripheral MDSCs, which play an important role in immune escape through generation of the so-called metastatic niche. Tumor-derived OPN was found to enhance both extramedullary myelopoiesis and the subsequent accumulation of MDSCs [96]. Silencing of OPN in tumor cells delayed both tumor growth and extramedullary myelopoiesis, whereas treatment with an antibody against OPN inhibited tumor growth-mediating antitumor immunity. Recently, an interesting study on a spontaneously metastatic model of breast cancer has shown distinct and common activities of OPN when produced either by tumor or host cells. Tumor-produced sOPN supports cancer cell survival in the blood stream, whereas both tumor- and host-derived OPNs, particularly from myeloid cells, render the metastatic site more immunosuppressive, thanks to the expansion of MDSCs at both the primary and lung metastatic sites. OPN was produced mainly as sOPN by cancer cells and as iOPN by myeloid cells, which, as described earlier, are both involved in cancer dissemination and play a pivotal role in inducing immunosuppression in the metastatic niche [96].

#### 8.1.3. Bone Marrow-Derived Cells

A new role for tumor-derived OPN has been ascribed to the activation and mobilization of bone marrow-derived cells to the microenvironment of disseminated tumor cells, the so-called “pre-metastatic niche” [146]. McAllister et al. found that the secretion of soluble OPN by a tumor supports stimulation of distant tumor/metastatic cells, otherwise indolent. This systemic “instigation” is accompanied by incorporation of bone marrow cells into the stroma of the distant, once indolent tumors, conditioning that environment and promoting tumor growth. This effect may represent a distinct pathophysiological role for circulating OPN in the blood of cancer patients [146]. Secretion of OPN by instigating tumors is necessary for bone marrow-derived cell activation and the subsequent outgrowth of the distant, otherwise indolent tumors. Overall, these results reveal that outgrowth of indolent tumors can be governed on a systemic level by endocrine factors released by certain instigating tumors and therefore hold important experimental and therapeutic implications.

### 8.2. Enhanced Invasion and Angiogenesis

Tumor-derived OPN may also play a role in angiogenesis. The proof of concept has been demonstrated brilliantly by many investigators. Proliferation, migration, and tissue infiltration of pericytes, vascular smooth muscle, and endothelial cells from preexisting blood vessels are needed for tumor angiogenesis, and OPN may participate in all of these processes. [166–169]. The role of OPN in tumor angiogenesis is associated with VEGF as both are frequently and simultaneously upregulated during angiogenesis [170–173]. In the preclinical model, OPN stimulates angiogenesis by inducing VEGF expression in endothelial cells [174]. OPN itself can be upregulated by fibroblast growth factor- (FGF-) 2 in endothelial cells in vitro and in vivo, leading to the recruitment of proangiogenic monocytes to the tumor microenvironment [175]. Interestingly, our group has recently shown that thrombin-cleaved OPN generates two fragments, namely, OPN-N and OPN-C, which display a stronger angiogenic potential in vitro, compared to full-length OPN [176]. These results are in line with the reports from Senger et al. [177] showing that VEGF induces OPN and *α*v*β*_3_ expression in endothelial cells and stimulates cleavage of OPN by thrombin and that the resulting OPN fragments are strongly chemotactic for endothelial cells and promote angiogenesis [178]. However, these authors used a mixture of the two OPN fragments obtained by thrombin-mediated cleavage of OPN-FL in vitro. Therefore, they could not distinguish the specific contributions of OPN-N versus OPN-C. Moreover, other studies have shown that, in vascular endothelial cells, OPN enhances VEGF-*α* expression, which, in turn, mediates a positive feedback on OPN expression; blocking this feedback signal by anti-VEGF-*α* antibodies partially inhibited OPN-induced HUVECs motility, proliferation, and tube formation [174].

### 8.3. Epithelial to Mesenchymal Transition

Fibroblasts are the predominant cell type in stromal connective tissue contributing to deposition and maintenance of collagen, basement membrane, and paracrine growth factors. As mentioned earlier, CAFs originate from different sources including local fibroblasts and MSCs recruited from the bone marrow and thus become specialized stromal cells with myofibroblast features able to promote tumor growth and dissemination by stimulating angiogenesis, cancer cell proliferation, and production of tumor-promoting cytokines [179, 180]. Additionally, CAFs can become a critical component of the cancer stem cell (CSC) niche, thereby favoring cancer metastasis, drug resistance, and disease relapse after chemotherapy regimens. Recently, it has been shown that breast cancer-derived sOPN can educate mammary fibroblasts to become proinflammatory CAFs, thereby favoring malignant progression [181]. Indeed, neutralizing antibodies against OPN blocked fibroblast reprogramming elicited by these malignant cells. Strikingly, OPN silencing in tumor cells not only attenuated stromal activation but also inhibited tumor growth, indicating once more that OPN plays a key role in reprogramming normal fibroblasts into tumor-promoting CAFs. Alternatively, CAFs can also be formed in response to OPN-induced MSC-to-CAF transformation [180]. One of the most important effects exerted by OPN following CAF activation is the modulation of tumor-specific EMT through the secretion of TGF-*β* and IL-6. In this regard, the transcription factor myeloid-zinc finger 1 (MZF1), activated through protein kinase A signaling, appears to be a critical mediator of this process [182]. Besides MSCs, tumor-derived OPN can also convert normal mammary fibroblasts into CAFs using in vitro and in vivo models of breast cancer [179]. Intriguingly, OPN bound to cell-surface integrin receptors activated MZF1, which in turn mediated TGF-*β*1 production by MSCs. Remarkably, aptamer-mediated inhibition of OPN binding to integrin receptors abolished this MZF1- and TGF-*β*-mediated MSC-to-CAF transformation. Since the adoption of the CAF phenotype is associated with increased local tumor growth and metastases, it is likely that therapeutic tools able to disrupt this pathway might be an alternative treatment option to current breast cancer therapy.

### 8.4. Mesenchymal to Epithelial Transition

MET is a reversible biological process that involves the transition from motile, multipolar mesenchymal cells to planar arrays of polarized cells called epithelia. MET is the reverse process of EMT. METs occur in normal development, cancer metastasis, and induced pluripotent stem cell reprogramming. In particular, MET is believed to participate in the establishment and stabilization of distant metastases by allowing cancerous cells to regain epithelial properties and integrate into distant organs [182]. For this reason, in recent years, MET has been regarded as one of many potential therapeutic targets in the prevention of metastases.

iOPN, the intracellular form of OPN mentioned earlier in this review, was initially described in rat calvarial cells by Zohar et al. [183], respectively; OPN intracellular immunostaining shows four distinct patterns: perimembranous staining, nuclear retention, cytoplasm distribution, and perinuclear staining, which represents sOPN. Mounting evidence has revealed crucial roles of iOPN in a number of processes contributing to cancer progression and dissemination, such as migration, cell cycle, and motility [76, 78, 184]. Recently, a role for iOPN in cancer metastasis has been proposed by Jia et al. [147]. Early in the metastatic cascade, cancer cells from the primary tumor undergo EMT, which endows noninvasive tumor cells with the ability to invade and disseminate [182, 185]. In recent years, MET has been shown to contribute to colonization at a secondary site. Recent findings support the hypothesis of sOPN and iOPN having distinct roles in phenotypic plasticity during different stages of tumor metastasis. In fact, sOPN promotes EMT to initiate early metastatic dissemination, whereas iOPN induces MET to facilitate metastatic colonization at later stages of metastatic colonization.

## 9. Effect of Stroma-Derived sOPN on the Tumor

In cancer, the coordinated intercellular interactions that are present in normal tissues are disrupted as the tumor acquires the ability to chronically circumvent physiologic signals from the microenvironment, which in turn evolves to accommodate the growing tumor [114, 186, 187]. As illustrated in the previous section, cancer is a heterogeneous disease involving genetic mutations in tumor cells. Nevertheless, it appears more and more evident that tumors are also diverse according to the nature of their ECM composition and stromal cell proportions or activation states [121, 188]. In response to evolving conditions and oncogenic signals from tumors, the stroma continually changes over the course of the entire cancer evolution. This has led to consider the influence of the microenvironment on metastasis as a dynamic process and prompted many investigators to determine how tumor cells drive the construction of their own niche. This aspect is particularly important in view of the fact that in-depth knowledge of mechanism of tumor stroma-induced neoplastic progression may provide additional therapeutic options to treat malignant carcinomas. In this regard, it has been shown, for example, that stroma cells not only can exert a protumorigenic effect in some solid tumors but can also be reprogrammed by pharmacological agents to exhibit antitumor activities [189].

### 9.1. Tumor-Associated Macrophages

As mentioned earlier, OPN-mediated regulation of macrophage functions has profound consequences in terms of tumor development. Nevertheless, also TAMs secrete OPN that can mediate protumorigenic effects. In this regard, a potential role of TAM-derived OPN in regulating CSC functions has been proposed [190]. CSCs are rare immortalized cells within the tumor characterized by self renewal, which can give rise to many cell types that can either constitute parts of the tumor or form new tumors. Since CSCs are ubiquitously expressed in a wide range of human cancers, they represent attractive targets for chemotherapy. A few reports have shown that TAMs interact with CD44^+^ colorectal CSCs by secreting OPN, thus promoting CSC tumorigenicity. Interestingly, OPN secretion by TAMs is stimulated by CD44^+^ colorectal cancer cells, and the induction of OPN is closely associated with CD44 expression. Although the exact mechanism whereby CD44^+^ cancer cells stimulate OPN secretion is not clear, this study suggests that, despite strongly relying on their niche, CSCs can reprogram stromal cells (e.g., TAMs) so that the latter can gain a growth advantage.

#### 9.2. Senescent Fibroblasts

Cancer incidence increases with aging and is associated with tissue accumulation of senescent cells [191]. A vast body of literature points to the fact that senescent fibroblasts contribute to tumor development in aging tissues [192–196]. OPN is indeed necessary for the promotion of preneoplastic cell growth by senescent fibroblasts [197]. Furthermore, senescent fibroblasts stimulated the growth of preneoplastic keratinocytes both in vitro and in vivo using mouse model of skin tumor. Silencing of OPN did not prevent stress-induced senescence in fibroblasts but rather blocked their ability to induce cell growth in associated keratinocytes [198]. An important effect of OPN on senescent fibroblasts relies on its crosstalk with Tiam1. Tiam1 is a Rac exchanger, ubiquitously expressed and involved in a number of signaling pathways [199–202]. In cancer cells, Tiam1 expression plays a key role in favoring tumor growth [203–205], and its expression in the stroma controls tumor invasion. OPN is a major mediator of the effects of Tiam1 expression in fibroblasts undergoing stress-induced senescence [191]. Stress-induced senescence in fibroblasts induces decreased fibroblast Tiam1 and increased OPN expression and secretion. Altering Tiam1 expression in CAFs induces changes in invasion, migration, epithelial-mesenchymal transition, and cancer stem cell characteristics in associated breast cancer cells. Interestingly, these changes persist even after cancer cells have dissociated from the fibroblasts. Altogether, these findings suggest that promalignant signals from the tumor stroma, with long-lasting effects on associated cancer cells, may sustain the metastatic potential of developing cancers. Thus, inhibition of these microenvironment signals may represent a new therapeutic strategy against cancer metastasis. These novel therapeutic tools will most likely be based on specific targeting of stromal cells, which display less genetic plasticity than their malignant counterpart.

#### 9.3. Natural Killer Cells

NK cells are innate lymphoid cells, which play an important role in mediating the anticancer immune response. These cells can survey and control tumor initiation due to their ability to recognize and kill malignant cells and to regulate the adaptive immune response via cytokines and chemokines release. However, several studies have shown that tumor-infiltrating NK cells associated with advanced disease can have profound functional defects and display tumorigenic activity. The role of iOPN was recently investigated also in NK cells by Leavenworth et al. [26], who demonstrated that NK cells require iOPN to prevent apoptosis and subsequently reach their full maturation and lytic activity. They used a mouse model where melanoma cells were coinjected with either NK cells unable to produce OPN (OPN-KO) or NK cells able to express only iOPN (iOPN-KI) into lymphopenic Rag^−/−^*γ*C^−/−^ mice. Mice reconstituted with iOPN-KI NK had an increased number of NK cells and a reduced metastatic dissemination. Thus, iOPN plays an important role in the formation of long-lived NK cells with a memory-like phenotype. Nonetheless, it remains to be determined whether iOPN is involved in the generation of antigen-driven memory NK cells and if it can exert a similar effect on memory T cells [26].

OPN is generally thought to display both protumorigenic and prometastatic functions. Nevertheless, a few reports have demonstrated that OPN also inhibits tumor progression. In particular, OPN-deficient mice accelerated tumor growth in a squamous cell carcinoma model [159, 206], and OPN-deficient macrophages showed impaired antitumor cytotoxicity [206]. Consistently, a recent report has shown that stroma-derived OPN enhances infiltration of NK cells into the transgenic adenocarcinoma of mouse prostate (TRAMP) tumors [207]. The requirement of OPN in NK cell migration towards tumor cells was confirmed by an ex vivo cell migration assay. The observation that the antitumorigenic function of OPN was evident only in Rag2−/− mice indicates that cells coordinating the adaptive immune response are not essential for OPN-mediated inhibition of TRAMP tumor development. Surprisingly, B16 melanoma tumor development was not affected by OPN, and B16 tumors did not show OPN-mediated cell recruitment. These results suggest that the antitumorigenic functions of OPN are tumor-type specific [207].

## 10. Osteopontin in Hematopoietic and Lymphoid Tumors

OPN role in several forms of non-Hodgkin lymphomas (NHL) and in acute leukemias has also been investigated, especially in recent years. OPN gene is upregulated in primary central nervous system lymphomas (PCNSL) as compared to that in diffuse large B cell lymphoma (DLBCL) [208], and its levels in cerebrospinal fluid (CSF) appear to be an independent predictor of shorter progression-free and overall survival [209]. Serum levels of OPN also predict response to therapy and survival in DLBCL [210, 211] and serum and CSF levels were correlated to tumor bulk and response to therapy in children with acute lymphoblastic leukemia (ALL) [211]. Expression of OPN is increased in both bone marrow blasts and serum of patients with acute myeloid leukemia (AML), and high-OPN mRNA expression independently predicts overall survival (*p* = 0.025), especially in low-intermediate risk forms [212]. Taken together, these elements suggest that OPN represents a potential biomarker for several lymphoproliferative forms [213].

From a pathogenic point of view, OPN is involved in invasion and dissemination of CNS lymphomas through activation of NF-*κ*B, an effect mediated by both iOPN (transcriptional downregulation of NF-*κ*B inhibitors) and sOPN (receptor-mediated activation of NF-*κ*B). NF-*κ*B can then induce MMP-8 and other MMPs, a pivotal mechanism of neoplastic tissue invasion and metastasis in human cancers [214]. Consistently, OPN was independently associated with increased MMP levels and higher circulating levels of OPN, MMP-2, and MMP-9 and were detected in patients with several forms of NHL as compared with those in healthy donors [215]. Overall, available data suggest that OPN may be involved in selective CNS tropism of lymphoma cells leading to PCNSL [216]. OPN has also been involved in resistance to chemotherapy in ALL, by anchoring leukemic blasts to endosteal niche within the bone marrow, supporting cell cycle exit and tumor dormancy; inhibition of OPN increases Ki-67 proliferative index, enhancing response to Ara-C chemotherapy [217]. Of note, OPN genetic polymorphisms appear to modulate sensitivity to Ara-C in a Chinese population with AML [50]. Acquired expression of OPN promotes enrichment and survival of leukemic stem cells (LSC) through the AKT/mTOR/PTEN/*β*-catenin/NF-*κ*B signaling pathways in AML [212]. Conversely, silencing OPN with specific sRNA appears to decrease colony numbers of LSC [218].

Overall, the multifaceted involvement of OPN in etiopathogenetic mechanisms of different forms of lymphomas and acute and chronic leukemias could lend support to a variety of potential therapeutic approaches.

## 11. Therapeutic Strategies Targeting OPN in Cancer

The pleiotropic effects of OPN in promoting tumor growth and metastasis and the close relationship between patient death and OPN expression render this protein an interesting target for cancer therapy [219]. Furthermore, as the review described before, an important issue concerning OPN is that it is secreted not only by tumor cells but also by several cells of the stroma, that are genetically stable compared to tumor cells and are thus supposed to be protected by classical mechanisms of pharmacologic resistance. In this regard, different OPN-targeting strategies have been proven effective in preclinical models, including OPN gene silencing, OPN receptor blockage, or inhibition of OPN posttranslational modifications such as thrombin cleavage and transglutamination [28, 220].

### 11.1. Inhibition of OPN by RNA Interference

Early experiments showed that it was possible to inhibit growth of osteosarcoma and oral cancer cells by blocking OPN mRNA translation [221, 222]. However, since the advent of RNA interference (RNAi), a number of studies have reported a much more efficient inhibition of OPN compared to traditional methods.

Indeed, RNAi is a biological process whereby RNA molecules inhibit gene expression or translation by neutralizing targeted mRNA molecules [223]. Essentially, three types of small RNA molecules, namely, microRNA (miRNA), small interfering RNA (siRNA), and short hairpin RNA (shRNA), are able to direct sequence-specific gene inhibition in mammalian cells [224]. As mentioned above, the encouraging preclinical data obtained using this innovative approach have prompted several investigators to conduct a number of ongoing clinical data, with the hope to provide proof-of-concept evidence that siRNA-, shRNA-, and miRNA-mediated inhibition of OPN exerts an antitumorigenic effect [225]. This is particularly true for breast cancer, as silencing of OPN not only inhibited cancer progression through downregulation of uPA, MMP-2, and MMP-9 expression levels [226] but also rendered these cells more sensitive to radiation-induced apoptosis and senescence [227] and enhanced their sensitivity to chemotherapeutic agents [228]. Although these results suggest that silencing of OPN might represent a promising strategy for the development of effective anticancer agents, one cannot help thinking that OPN-directed RNAi would inevitably target both the secreted and intracellular forms of OPN. Thus, given the opposite role of these two proteins in cancer progression, as described earlier in this review, it is likely that selective inhibition of one form versus the other might prove more effective in reducing the tumor burden. In this regard, the use of blocking antibodies against sOPN or its receptor selectively inhibited sOPN functions while preserving those of iOPN.

### 11.2. Inhibition of OPN by Blocking Antibodies

As described above, OPN binds to two sets of receptors, namely, integrins and CD44, which then propagate downstream signaling. Thus, an additional way to inhibit OPN can be readily achieved by using blocking antibodies specific for of one or both receptors. Indeed, a number of antibodies targeting these receptors have been shown to significantly suppress the interaction between tumor and stroma, thereby reducing OPN-induced tumor progression. For example, blocking OPN binding to *α*v*β*3 inhibited OPN-induced tumor growth and angiogenesis [139], decreased the expression of ILK, uPA, and MMP-2 [229], and prevented OPN-mediated AP-1 activation in breast cancer cells [230]. Accordingly, an anti-OPN antibody and its humanized version effectively inhibited tumor growth and angiogenesis in a breast cancer model [174]. Recently, a new integrin-binding site has been identified in the C-terminal fragment of MMP-3/7-cleaved mouse OPN, binding to the *α*9*β*1 integrin. Importantly, this novel motif is involved in the development of anti-type II collagen antibody-induced arthritis (CAIA), and blocking OPN interaction with *α*9*β*1 prevents CAIA [86]. Furthermore, given that *α*9*β*1 integrin contributes to tumor growth, lymphatic metastasis, recruitment of CAFs, and induction of OPN secretion by CAFs, inhibition of the *α*9*β*1 integrin-OPN axis protected mice also from breast cancer [231].

With respect to CD44, it is well known that such receptor varies in size due to glycosylation and alternatively spliced exon products (i.e., CD44v) [232] and that these CD44 variants are often overexpressed in cancer cells and metastasis [233]. For example, CD44v6-7 is able to bind OPN [234] and it is overexpressed in tumor-infiltrating leucocytes in colorectal cancer [235] and in malignant melanoma [236]. CD44 blockade leads to two major effects in the preclinical model: higher number of macrophages and strong increase of OPN production inside the tumor. Aptamers are short oligonucleotides or peptides able to specifically bind to small molecules or protein ligands by forming a three-dimensional structure complementary to the target molecules [237]. Aptamers are functionally comparable to traditional antibodies but offer several advantages such as their relatively small physical size, flexible structure, quick chemical production, high stability, and resistance to immunogenicity. Furthermore, they are effective at very low concentrations, thus offering an important advantage over antibody-mediated therapy. Thus, OPN has also been targeted in cancer by the means of aptamers. OPN-directed RNA aptamer (OPNR3) binds specifically to OPN and decreases in vitro cellular adhesion, migration, and invasion in breast cancer cells [238]. Upon extensive pharmacokinetic characterization of OPN-R3 aptamer, Talbot et al. demonstrated the efficiency of modified OPN-R3 aptamer in suppression of breast tumor growth [239].

### 11.3. Inhibition of OPN by Targeting Its Posttranslational Modifications

Among the different PTMs of OPN, two, namely, thrombin cleavage and transglutamination, appear to be an attractive target for cancer therapy. In this regard, Schulze et al. have shown that the thrombin inhibitor argatroban inhibits both tumor growth and lymphatic metastasis occurrence of breast cancer cells by blocking the formation of OPN thrombin-cleaved fragments [240]. A second therapy that may alter carcinogenesis secondary to OPN consists of transglutaminase inhibitors [241]. Tissue transglutaminase catalyses bond formation between glutamine and lysine of two side chains, thus inducing the cross-linking of proteins. This process is essential for the stabilization of the ECM and can be deregulated during cancer metastasis. OPN is a target of transglutaminase and its polymerisation induces some gain of functions [242]. Several transglutaminase inhibitors exist that have been proven effective in cancer [243].

### 11.4. Anti-OPN Autoantibodies (autoAbs)

Antibodies anti-OPN are spontaneously produced in several conditions in the presence of high circulating OPN levels as in the case of MS [244], RA [245], and more recently HCC [246]. In the latter case, the titer of anti-OPN autoantibodies in HCC was significantly higher than in healthy human serum. The authors went on showing that this increase in OPN autoantibody production correlated with poor prognosis and could therefore be considered a new bona fide serological biomarker for HCC. Since our group has shown that OPN protein vaccination of mice predisposed to multiple sclerosis was effective in inducing a neutralizing antibody response that reduced OPN levels while protecting these mice from disease occurrence, it is possible that a similar vaccine-based strategy may prove effective for cancer therapy as well.

### 11.5. Small Molecule Protein-Protein Interaction (PPI) Inhibitors

PPIs influence biological functions by modulating protein activities, such as enzymatic activity, subcellular localization, and/or binding properties. Interfering in PPIs is considered to be a promising strategy towards next-generation therapeutics, including those for cancer [247]. Inhibition of PPIs by mean of small molecules is now recognized as an emerging and challenging area in drug design.

Recently, Park et al. identified a novel small molecule inhibitor, IPS-02001, targeting the integrin *α*v*β*3-OPN PPI, by using in silico docking method-integrated ProteoChip technology. They tested its biological function in vitro and demonstrated that it was efficient in inhibiting OC maturation and resorptive function by blocking integrin signaling, which disrupts actin cytoskeletal organization. In vivo, IPS-02001 blocked RANKL-induced bone destruction and suppressed ovariectomy-induced bone loss. This pioneering work showed that IPS-02001 is a potent inhibitor of integrin-mediated OPN signaling and suggests that it may be used also in cancer [248].

## 12. Conclusions

OPN is overexpressed in a variety of human carcinomas and has been implicated in inflammation, tumor progression, and metastasis. Within the tumor microenvironment, OPN can be produced by many cell types including the tumor itself and stromal cells. Most therapeutic strategies against cancer have focused on direct targeting of various tumor cell features. However, stromal cells and their ECM are genetically stable compared to tumor cells and are therefore supposed to be less susceptible to classical mechanisms of pharmacologic resistance. For all these reasons, OPN is regarded by many as one of the most attracting targets for cancer therapy. Nevertheless, targeting OPN for therapeutic purposes will have to take into account the heterogeneous functions of the multiple OPN forms with regard to cancer formation and progression. These functions can be either protumorigenic or antitumorigenic according to cell type and tumor microenvironment. Moreover, literature showed several conundrums suggesting that knowledge on OPN is only at the tip of the iceberg and that new functions and possibly binding partners may exist, further complicating the scenario.

Although OPN represents an extraordinary interesting potential target for cancer therapy, we still need to be cautious for a number of reasons. First, the several forms of OPN may have distinct effects in different tissues and tumors. Therefore, new insights are needed to depict these differences and to setup approaches targeting distinct OPN forms and activities and distinct cells and tissues. Second, it must be noted that some reports suggest that OPN may exert antitumorigenic activity in some instances. Finally, therapies targeting OPN might share the limitations of other immunotherapies which, despite positive preclinical achievement, fail to reach satisfying therapeutic effects because of development of tumor resistance, redundant effects displayed by similar molecules, and adverse side effects due to the multiple pleiotropic activities of OPN.

## Figures and Tables

**Table 1:** Summary of several features of the main OPN isoforms.

	Isoform	Exons	aa differences	mRNA	Putative differences	NCBI ref seq
sOPN	OPN-a	2–7	Full length	All exons are translated	Full-length protein	NM_001040058.1
	OPN-b	2–7 Δ5	aa 59–72 are missing	Alternative splicing of exon 5	Less-phosphorylated domains	NM_000582.2
	OPN-c	2–7 Δ4	aa 31–57 are missing	Alternative splicing of exon 4	Less-phosphorylated domains + lack transglutamination signal	NM_00140060.1

iOPN	—	2–7 Δ signal peptide	aa 1–16 are missing	Downstream initiation of translation	Lack of signal peptide, intracellular localization	[73]
